# Unlocking the role of Smith-specific regulatory T-cells: a paradigm shift in autoimmune therapy

**DOI:** 10.1097/MS9.0000000000002449

**Published:** 2024-08-07

**Authors:** Shahood Ahmed Umar, Zahra Sania, Lamiya Pirzada, Sanila Mughal, Muhammad Umair Anjum, Mohammed Mahmmoud Fadelallah Eljack

**Affiliations:** a Department of Medicine, Ziauddin Medical University; b Department of Medicine, Jinnah Sindh Medical University; c Department of Medicine, Dow University of Health Sciences, Karachi; d Department of Medicine, Women Medical and Dental College, Khyber Medical University, Peshawar, Pakistan; e Faculty of Medicine and Health Sciences, University of Bakht Alruda, Ad Duwaym, Sudan

## Introduction

Systemic lupus erythematosus (SLE) is a recurrent and remitting autoimmune disease that affects 1.5 million people in the United States and is linked with significant morbidity and mortality. It is one of the main causes of death in females, predominantly targeting individuals between the ages of 16 and 55, with females to males at a ratio of 9 to 1^1^. Higher incidence of the disease has also been reported among the Black, Caribbean, Asian and Hispanic populations^2^, indicating varied susceptibility across diverse demographics. SLE exhibits multiple phenotypes, showing a spectrum of clinical presentations ranging from mild involvement of skin and mucosa to severe disease of multiple organs, including the central nervous system. The most prevalent and critical solid-organ manifestation in SLE is lupus nephritis (LN)^3^, which typically develops within the first 5 years following the SLE diagnosis^4^. The progression of LN often involves periods of flare-ups, marked by increased disease activity, interspersed with times when the disease is less active, which eventually leads to the gradual loss of nephrons and a steady deterioration of kidney function. Therefore, it is projected that between 5 and 20% of those with LN will advance to end-stage renal disease (ESRD) within a decade of being diagnosed with SLE, necessitating either dialysis or a kidney transplant^4^.

## Pathogenesis of systemic lupus erythematosus

The development of SLE involves intricate processes and our comprehension of its pathogenesis continues to evolve. The major antibodies responsible for its pathogenesis are the anti-nuclear antibodies (ANA), anti-double-stranded DNA antibodies (anti-dsDNA), and the anti-Smith antibodies, the latter carrying 99% specificity for the disease^2^. Certain auto-antibodies like anti-Ro (SSA) and anti-La (SSB) targeting ribonucleoprotein particles, otherwise common in Sjogren’s syndrome, have also been reported in patients suffering from lupus. In SLE, these antibodies cause a variety of symptoms such as dry eyes (keratoconjunctivitis sicca), skin rashes (subacute cutaneous lupus), and heightened photosensitivity. They may also lead to certain complications such as newborn lupus and congenital heart-block. Moreover, anti-histone antibodies are a hallmark of drug-induced lupus, which is mostly triggered by pharmacological agents such as hydralazine, procainamide, and isoniazid^2^. Thus, the anti-smith antibodies distinguished by their remarkable specificity of 99% provide a basis for developing targeted therapeutic approaches for SLE.

## Current treatment strategies

Despite the various treatment strategies used by physicians to treat SLE, it is challenging to standardize the patient treatment and SLE is considered incurable^5^. Varied clinical manifestations influenced by genetics, socioeconomic, and environmental factors further complicate the management strategies^6^. The European Alliance of Associations for Rheumatology recommends a treat-to-target principle that entails controlling disease activity and preventing end-organ damage and flares on the skin to minimize the severity of SLE^7^. Therefore, the main modality of treatment for SLE includes anti-inflammatory and immunosuppressant medications like glucocorticoids, non-corticosteroids, immunosuppressants, and antimalarial drugs^5^. Several biological agents including anifrolumab, belimumab, and rituximab have been approved for treating persistently active or recurrent SLE. However, increased rates of infectious complications including bronchitis, nasopharyngitis, herpes zoster, and urinary tract infections have been reported in patients treated with these biologic agents. Similarly, calcineurin inhibitors and Bruton’s tyrosine kinase inhibitors have indicated successful results in treating autoimmune abnormalities, but their selectivity and toxicity have limited their use^8–10^. These limitations highlight the need to develop effective and innovative treatment approaches to advance toward a potential cure for SLE.

## The role of Smith-specific regulatory T-cells

SLE stands as a classical prototype in the world of systemic autoimmune diseases as it is widely accepted that an overall reduction in Tregs (regulatory T-cells) or deterioration in their function tends to exacerbate SLE pathology. Antigen-specific Tregs, a specialized group of T-cells that express the IL-2 receptor α-chain (CD25), were initially recognized for their role in preventing systemic autoimmune diseases by preserving self-tolerance. To accomplish this, they either release anti-inflammatory mediators like IL-10 and TGF-β or directly interact with cells to suppress the stimulation and proliferation of self-reactive lymphocytes^11^. Thus, the involvement of antigen-specific Tregs in reducing autoimmune responses provides a positive signal to their therapeutic potential in SLE.

Horwitz *et al.* ^12^ discussed how nanoparticles can be used to regenerate therapeutic antigen-specific Tregs as well as stimulate antigen-presenting cells to become protective Tregs instead of pathogenic T-cells. Ensuring the resurgence and sustenance of Treg dominance over effector cells using this method may foster prolonged remission from autoimmune diseases. However, there are substantial hurdles in creating Nano particle-based therapies as well because the autoimmune presentation may vary from person to person so the therapeutic effects will vary as well. Secondly, scaling up laboratory formulations of therapeutic nanoparticles to achieve clinical-grade quantities will pose a significant challenge as well^12^. In addition, a 2014 study^13^ sought to develop a secure and effective strategy for antigen-specific immunotherapy. Examination of the transcriptome of CD4+ T-cells across various phases of progressively higher dose immunotherapy indicated a gradual decrease in transcripts that promoted inflammatory effector function, along with suppression of cell cycle pathways. Transcription factors c-Maf and NFIL3, as well as negative co-stimulatory substances LAG-3, TIGIT, PD-1, and TIM-3, were discovered to identify this regulatory CD4+ T-cell fraction, and their level of expression are correlated with the immunoregulatory cytokine IL-10. The findings indicated novel transcriptional and immunological signatures as markers for effective immunotherapy. In their study, Clemente *et al.* ^14^ demonstrates that the introduction of nanoparticles covered with peptides relevant to autoimmune diseases, linked to the molecules of major histocompatibility complex class II (pMHCII), induces the production and proliferation of CD4+ T-cell type 1 (TR1)-like cells in various murine specimens resulting in the alleviation of existing autoimmune manifestations. Ten pMHCII-based nanomedicines possess identical biological consequences thus encouraging the transformation of disease-primed self-reactive T cells into TR1-like cells, which subsequently inhibit autoantigen-loaded antigen-presenting cells and facilitate the development of specific B cells into regulatory B cells that suppress the disease, while maintaining systemic immunity. Hence these nanomedicines based on pMHCII could be beneficial for addressing a wide range of autoimmune disorders including SLE with a targeted approach tailored to each specific disease.

**Figure 1:**
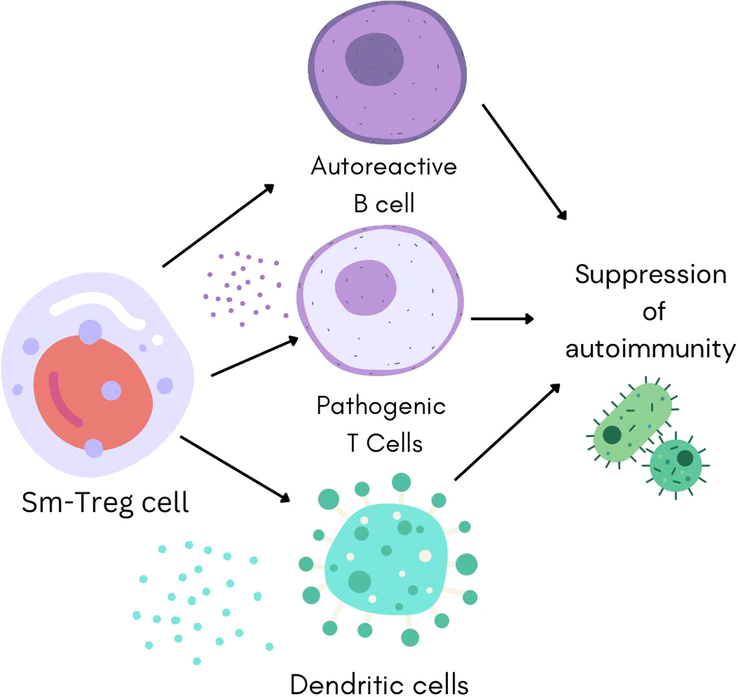
Mechanism of action of Smith-specific regulatory T-cell.

Nevertheless, as reported, Human leukocyte antigen (HLA)-DR15 haplotype and Sm autoantigen have a connection with LN, therefore, Eggenhuizen *et al.* ^15^ experimented to explore the potential of Smith-specific Tregs to curb autoimmune responses. The researchers used biophysical affinity binding assays to detect a HLA-DR15 restricted immunodominant Sm T-cell epitope and then Sm-specific Treg receptors (Sm-TCRs) were identified using high-throughput single-cell sequencing. The Sm-TCRs were then transformed into Tregs using lentiviral vectors which were derived from anti-Sm and HLA-DR15 positive SLE patients. The results showed that in humanized murine specimens of LN, Sm-Tregs strongly inhibited both disease progression and Sm-specific pro-inflammatory reactions *in vitro* as compared to polyclonal mock-transduced Tregs. Using the results of this experiment as a foundation, the researchers created a platform for developing TCR Treg-cell-oriented treatments that can be expanded to combat additional clinical symptoms of SLE, including the selective targeting of diverse autoantigens and HLA-types, as well as other autoimmune diseases associated with HLA^15^. The mechanism of action of Sm-Tregs has been illustrated in Figure 1.

Although the previous methods hold potential for the treatment of SLE, this recent research regarding Sm-Tregs holds high potential for the treatment because it has demonstrated that when transduced onto primary Tregs from patients, the Sm-TCRs impart Smith specificity and the capacity to effectively block Smith self-reactivity in-vivo as well as *in vitro*. The protein crystal structure of SmB/B’58-72 bound to HLA-DR15 confirms that SmB/B’58-72 interacts with HLA-DR15 in a typical way. This structure is one of only three known structures of peptides binding to HLA-DR15 for human self-antigens. Understanding how specific parts of the self-antigen bind to HLA-DR15 will help in designing better TCRs for LN and other diseases linked to HLA analysis will also aid in identifying potential off-target effects of new treatments because we now understand which regions of self-antigens Sm-TCRs can identify. The significant rise in CD52 levels seen in TCR1-expressing cells suggests a possible correlation with a particular subset of suppressor T cells. The research also shows that IL-2 adjunct therapy is not required for Sm-Tregs as IL-2 supplementation *in vitro* is adequate for reinstating Treg function and facilitating sustained engraftment over time^15^.

## Development and implementation of Smith-specific T-regs

In the first step of developing smith-specific Tregs^15^, the immunogenic autoimmune epitope, the Sm antigen was recognized using a biophysical affinity binding assay. Among the Sm epitopes, SmB/B'58-72 showed the strongest binding and highest stability for HLA-DR15 of any Sm peptide tested. Furthermore, the top three Sm peptides were assessed for immunogenicity using an in-vitro proliferation assay, and SmB/B'58-72 elicited the greatest T-cell proliferation response. Therefore, SmB/B'58-72 was recognized as the dominant T-cell epitope in lupus nephritis. Furthermore, a protein crystal structure of SmB/B'58-72 in combination with HLA-DR15 was solved in which it was found that SmB/B'58-72 binds well to the P1 and P4 hydrophobic peptide binding pockets.

In the next step, SmB/B’58-72-specific T-cell receptors (TCRs) were recognized using a combination of high-throughput antigen-specific TCR single-cell sequencing, dextramer binding affinity assays, and imaging flow cytometry. The most prevalent TCR clonotype with the highest affinity for SmB/B'58-72, TCR1, had 59 clones, more than three times higher than the remaining two clonotypes, TCR2 (16 clones) and TCR3 (13 clones). Thus, the lead Sm-TCR was chosen to be TCR1, which demonstrated considerable clonal proliferation and functional activity, indicating a therapeutic potential.

Lentiviral vectors encoding TCR 1-3 were then synthesized to transduce the first line of T-cells called J76 jurkat T-cell line lacking endogenous TCR and primary human Tregs. Sm-TCR1 lentiviral vectors were later transduced into human Tregs taken from healthy human donors. The validity of Sm-TCR1 Tregs was checked by assessing their function both *in vitro* and *in vivo* in a humanized mouse model of lupus nephritis. These modified Tregs not only maintained their regulatory function but also demonstrated increased specificity and suppressive activity against Sm epitope-induced pro-inflammatory responses.

Research regarding the mass production and methods of infusion of Sm-specific Tregs in humans is still lacking. However, the study by Eggenhuizen *et al.* ^15^. has shown promising results, which can be used as the basis for starting a clinical trial.

## Advantages of Smith-specific T-regs

This ground-breaking discovery for treating Lupus has long-term implications, which could eventually lead to its cure. One of the paramount advantages of this discovery is its specificity, that it is not going to harm the rest of the immune system and will only be used to cure SLE. This precision enhances its potential as a safe and effective SLE remedy^15^. Another major advantage is that the treatment not only targets and eliminates the harmful aspects of SLE but also actively strengthens the immune system’s defensive components which suppress autoimmunity thus curbing the harmful effects of this disease^16,17^. This dual action enhances the body’s defense mechanisms, developing an extensive and balanced immune response. Additionally, this discovery curbs the necessity for detrimental immunosuppressants and their need diminishes significantly. The breakthrough offers a viable alternative, sparing individuals from the adverse effects associated with traditional immunosuppressive therapies, thereby improving the overall quality of life for SLE patients. Not only does this cure protect the immune system but this innovative approach holds the potential to streamline medication regimens for SLE patients, replacing the need for numerous drugs. It has the potential to reduce the number of various medications that patients with SLE require. The singular treatment option signifies a transformative shift, simplifying healthcare and enhancing patient convenience and adherence.

## Challenges and limitations

While this discovery does have many positive aspects, there are also some challenges faced by it. The lentiviral (LV) system carries the risk of inducing insertional mutagenesis and insertional oncogenesis; if inserted inside exons^18^. Thus, further research should focus on and validate the provision of efficient LV delivery. Furthermore, there is a lack of information on the cross-reactivity of Sm-Tregs with other autoantigens. As reported, the phenotype of transferred cells transitions to a non-therapeutic state in some cases that can suppress the therapeutic response in vivo^19^. All the above-mentioned risks warrant comprehensive pre-clinical and clinical testing to validate the safety profile of this novel therapy. A notable limitation of this discovery lies in uncertainty regarding its long-term efficacy as a cure for SLE. While promising in its specificity and immediate benefits, further research is imperative to assess the treatment’s durability over time. Long-term studies are essential to ascertain the sustained effectiveness and potential recurrence of SLE symptoms post-treatment.

Another major challenge is the careful development required for disease-specific treatments, considering the distinct nature of each ailment. This specialized approach, while ensuring precision, demands extensive time and resources. The necessity to customize treatments for various diseases may potentially elongate the implementation timeline, delaying the availability of treatment for autoimmune disorders such as SLE.

An additional challenge of this breakthrough is its medium-term efficacy, necessitating the potential for repeated treatments. While offering relief in the short to medium term, the necessity for periodic interventions raises questions about the long-term sustainability of the cure for SLE. Further research is essential to understand the frequency and implications of repetitive treatments for lasting effectiveness.

## Future implications

This novel therapy employing Sm T-regs could be used in combination with other immunosuppressant medications and biologic agents conventionally used in the treatment of SLE. Using combination therapy will help in reducing the doses of the administered immunosuppressants and biological agents that could potentially alleviate the adverse effects and toxicity reported with these drugs. Furthermore, the synergistic effects of the combined therapy will also enhance autoimmune suppression. Recent studies have shown that gut microbes provoke autoimmunity through mechanisms like translocation and molecular mimicry, which could lead to SLE development. Thus, strategies to modify microbiota such as pharmacological interventions, stool transplant, and dietary interventions along with administering Sm T-regs could also offer an enhanced cure for SLE^20^.

The far-reaching potential of this treatment extends beyond SLE, encircling a diverse range of diseases including Sjogren syndrome, scleroderma, myasthenia gravis, multiple sclerosis, diabetes mellitus and rheumatoid arthritis. Its adaptability hints at a revolutionary pattern in healthcare, promising novel therapeutic approaches. If successfully applied to these conditions, the treatment could usher in a new era of comprehensive and effective interventions, offering hope for improved outcomes and quality of life for individuals grappling with different autoimmune disorders.

Additionally, a GMP-ready closed processing system utilizing the Sm-Treg production method is being created. This system can be employed as a cell-based treatment in a phase I clinical trial for individuals with LN, providing a secure environment for treating it on human subjects^15^.

## Conclusion

In conclusion, the implications of Sm-Tregs represent a promising treatment approach that holds great potential to revolutionize the management of SLE as well as other autoimmune diseases. Its efficacy in the cure of SLE along with its remarkable safety profile makes it a novel alternative to the currently approved treatment options. Although it suppresses auto-reactivity with precision and efficacy, further studies and clinical trials on a larger varied population are crucial to fully validate its broader potential. Nevertheless, with this novel treatment approach, the future holds promise for a paradigm shift in the treatment and cure of SLE.

## Ethical approval

This article did not involve patients; therefore, no ethical approval was required.

## Consent

Informed consent was not required for this editorial.

## Source of funding

No funding was received to assist with the preparation of this manuscript.

## Author contribution

S.A.U.: conceptualization of the study, writing—original draft, writing—review and editing. Z.S.: conceptualization of the study, writing—original draft. L.P.: writing—original draft, writing—review and editing. S.M.: writing—original draft, writing—review and editing. M.U.A.: supervision, writing—review and editing. M.M.F.E. supervision, writing—review and editing. All authors have read and approved the final version of the manuscript. The corresponding author takes complete responsibility for the integrity and accuracy of the data.

## Conflicts of interest disclosure

The authors declare no conflict of interest.

## Research registration unique identifying number (UIN)

Not applicable.

## Guarantor

Mohammed Mahmmoud Fadelallah Eljack.

## Data availability statement

Data sharing is not applicable to this article as no new data were created or analyzed in this study.

## Provenance and peer review

Not commissioned, externally peer-reviewed.
